# Strong association between non alcoholic fatty liver disease (NAFLD) and low 25(OH) vitamin D levels in an adult population with normal serum liver enzymes

**DOI:** 10.1186/1741-7015-9-85

**Published:** 2011-07-12

**Authors:** Ilaria Barchetta, Francesco Angelico, Maria Del Ben, Marco Giorgio Baroni, Paolo Pozzilli, Sergio Morini, Maria Gisella Cavallo

**Affiliations:** 1 Department of Internal Medicine and Medical Specialties, Sapienza University of Rome, Italy; 2 Endocrinology and Diabetes, Department of Medical Sciences, University of Cagliari, Cagliari, Italy; 3 Endocrinology & Diabetes (CIR), University Campus Bio-Medico, Rome, Italy; 4 Microscopic and Ultrastructural Anatomy (CIR), University Campus Bio-Medico, Rome, Italy

## Abstract

**Background:**

Hypovitaminosis D has been recently recognized as a worldwide epidemic. Since vitamin D exerts significant metabolic activities, comprising free fatty acids (FFA) flux regulation from the periphery to the liver, its deficiency may promote fat deposition into the hepatocytes. Aim of our study was to test the hypothesis of a direct association between hypovitaminosis D and the presence of NAFLD in subjects with various degree of insulin-resistance and related metabolic disorders.

**Methods:**

We studied 262 consecutive subjects referred to the Diabetes and Metabolic Diseases clinics for metabolic evaluation. NAFLD (non-alcoholic fatty liver disease) was diagnosed by upper abdomen ultrasonography, metabolic syndrome was identified according to the Third Report of National Cholesterol Education Program/Adult Treatment Panel (NCEP/ATPIII) modified criteria. Insulin-resistance was evaluated by means of HOMA-IR. Fatty-Liver-Index, a recently identified correlate of NAFLD, was also estimated. Serum 25(OH)vitamin D was measured by colorimetric method.

**Results:**

Patients with NAFLD (n = 162,61.8%) had reduced serum 25(OH) vitamin D levels compared to subjects without NAFLD (14.8 ± 9.2 vs 20.5 ± 9.7 ng/ml, p < 0.001, OR 0.95, IC 95% 0.92-0.98). The relationship between NAFLD and reduced 25(OH)vitamin D levels was independent from age, sex, triglycerides, high density lipoproteins (HDL) and glycaemia (p < 0.005) and Fatty Liver Index inversely correlated with low 25(OH) vitamin D regardless sex, age and HOMA-IR (p < 0.007).

**Conclusions:**

Low 25(OH)vitamin D levels are associated with the presence of NAFLD independently from metabolic syndrome, diabetes and insulin-resistance profile.

## Background

Vitamin D is a lipophilic molecule essential to calcium and phosphate balance and osteo-metabolic system regulation. It is produced onto the skin through a UV-mediated reaction, then it is metabolized to its active 1α,25 (OH)_2 _form through two consecutive hydroxylations exerted by kidney and liver, respectively. In adult population, the prevalence of hypovitaminosis D is from 5% to 30% [1], but it reaches a peak of 75% in patients with metabolic syndrome (MS) [2]. Several evidences have shown a thigh link between serum vitamin D level, calcium homeostasis and the risk for cardiovascular disease [3,4]. Interestingly, vitamin D deficient individuals are more likely to develop alterations in glucose metabolism, such as impaired glucose tolerance, the metabolic syndrome and type 2 diabetes mellitus [5-7]. Based on these evidences, it can be hypothesized that vitamin D deficiency should not be considered an exclusive feature of patients with osteo-mineral disorders. Vitamin D is capable to reduce FFA-induced insulin resistance both in peripheral tissues and in hepatocytes [8]. Therefore, low serum vitamin D may predispose to intrahepatic lipid accumulation leading to NAFLD.

NAFLD is a pathological condition consisting in a spectrum of liver diseases due to macrovesicular accumulation of triglycerides within hepatocytes (hepatic steatosis). In developed countries, NAFLD is observed in 20-30% of the general population [9,10] and in 75% of type 2 diabetic patients [11,12]; necro-inflammatory activity and fibrosis coexist in the 2-3% of cases (non-alcoholic steatohepatitis, NASH) and may evolve in cirrhosis and liver failure in 20-25% of affected subjects [13-15]. Currently, NAFLD is considered one of the leading causes of cryptogenetic cirrhosis [16].

Recently, an increased risk of cardiovascular disease in patients with NAFLD has been also suggested [17], based on the strong association between NAFLD and MS [18].

Thus, NAFLD is historically considered the hepatic component of MS, attributable to insulin-resistance that increases NEFA (non-esterified fatty acids) release from adipose tissue into the bloodstream and favors their deposition into hepatocytes [19]. However, recent investigations hypothesized a primary role of fatty liver in determining insulin resistance and its consequences [20]. Therefore, aim of this study was to test the hypothesis of an association between hypovitaminosis D and the presence/degree of NAFLD.

## Methods

### Population under study

To these purposes, we studied 262 consecutive subjects referred to the Diabetes and Hepatology outpatient clinics of Sapienza University of Rome for suspected MS. To be eligible for the study, patients had to fulfil the following criteria: normal serum liver enzymes, no history of current or past excessive alcohol drinking as defined by an average daily consumption of alcohol < 30 g/die in men and < 20 g/die in women; negative tests for the presence of hepatitis B surface antigen and antibody to hepatitis C virus; absence of history and findings consistent with cirrhosis and other chronic liver diseases. All subjects had a complete work-up including a clinical examination, anthropometric measurements, laboratory tests and a liver US scan.

### Laboratory determinations

Study population underwent fasting blood sampling to assess blood glucose (FBG), glycosylated hemoglobin (HbA1c), total cholesterol, HDL-cholesterol, triglycerides, aspartate aminotransferase (AST), alanine aminotransferase (ALT), gamma-glutamyl transpeptidase (gamma-GT), alkaline phosphatase, nitrogen and creatinine by standard laboratory methods. Insulin was measured by radio-immuno-assay (ADVIA Insulin Ready Pack 100, Bayer Diagnostics, Milan, Italy), with intra- and inter-assay coefficients of variation < 5%. Low-density lipoprotein (LDL) cholesterol value was obtained using Friedwald formula. The homeostasis model assessment of insulin resistance (HOMA-IR) was calculated as previously described [21]. MS was defined according to modified NCEP ATP-III criteria [22] and diabetes mellitus according to ADA 2009 criteria [23].

### NAFLD evaluation

Liver ultrasonography (US) scanning was performed to assess the degree of steatosis. All US were performed by the same operator who was unaware of the aims of the study and blinded to laboratory values using an Esaote Medica apparatus equipped with a convex 3,5 MHz probe. Liver steatosis was scored semiquantitatively on a scale of 0-3; 0, absent; 1, mild; 2, moderate; 3, severe. Steatosis was graded according to Saverymuttu et al. [24] on the basis of abnormally intense, high level echoes arising from the hepatic parenchyma, liver-kidney difference in echo amplitude, echo penetration into deep portion of the liver and clarity of liver blood vessel structure.

We also evaluated the Fatty Liver Index (FLI), a clinical and metabolic correlate of NAFLD, in both patients and non-NAFLD subjects. FLI is expressed as a 0-100 range number; a value < 30 rules out and >60 rules in the presence of hepatic steatosis with a sensibility and specificity of 87% and 86%, though the following formula: FLI = (e ^0.953*loge (triglycerides) + 0.139*BMI + 0.718*loge (ggt) + 0.053*waist circumference - 15.745^)/(1 + e ^0.953*loge (triglycerides) + 0.139*BMI + 0.718*loge (ggt) + 0.053*waist circumference - 15.745^) * 100 [25].

### 25(OH) vitamin D measurement

In order to assess the calcitriol balance in our population, we measured serum 25(OH) vitamin D, the most stable circulating form of this molecule [26,27]. Blood samples were obtained in the same season and 25(OH) vitamin D was measured by a validated colorimetric method (LAISON, DiaSorin) on sera frozen immediately after separation and stored at -25°C for few days.

### Statistics

SPSS version 17 statistical package was used to perform the analyses. Student's T-test for continuous variables and χ^2 ^test for categorical variables were used to compare mean values between two independent groups. Because HOMA-IR, FBG, triglycerides, AST, ALT, GGT and alkaline phosphatase were skewed, we used natural logarithmic transformations of these variables before performing means comparison (Student's T-test), ordinal and multivariate regression analyses. Logistic regression was used to estimate the predictive value of 25(OH) vitamin D and metabolic parameters on the presence of NAFLD, considered as dichotomous variable. Ordinal regression was used to detect the association between predictor variables and presence and degree of NAFLD (0: absence, 1: mild, 2: moderate, 3: severe). FLI and 25(OH) vitamin D were analyzed as continuous variables. A multiple liner regression analysis was performed to investigate independent association between FLI (dependent variable) and clinical and biochemical parameters. The comparison of clinical/biochemical characteristics among different 25(OH) vitamin D quartiles was performed by means of ANOVA and Kruskal-Wallis analyses, as appropriate. Data are shown as mean ± standard deviation. For all the above, a p-value < 0.05 was considered statistically significant.

The study protocol was reviewed and approved by the Ethics Commettee of Policlinico Umberto I, Sapienza University of Rome and conducted in conformance with the Helsinki Declaration. Written consent was obtained from all patients before the study.

## Results

Out of the 262 consecutive subjects who underwent liver US examination, 162 (61.8%) were affected by NAFLD (43.0% mild, 39.2% moderate and 17.8% severe) and 100 were free from NAFLD or other liver diseases.

NAFLD and non-NAFLD subjects differed significantly in several parameters, such as BMI (p < 0.001), waist circumference (p < 0.001) HDL (p < 0.001) and triglycerides (p < 0.001). Clinical and biochemical characteristics of study population are summarized in Table 1.

**Table 1:** Clinical and biochemical characteristics of study population.

Parameter	NAFLD (n = 162)	No NAFLD (n = 100)	p-value
Age (years)	52.07 ± 8.18	49.81 ± 7.7	n.s.

Sex^ (M/F)	89/73	51/49	n.s.

BMI (kg/m^2^)	31.36 ± 5.49	25.87 ± 5.1	< 0.001

Waist circumference (cm)	105.94 ± 12.68	90.26 ± 18.10	< 0.001

SBP (mmHg)	132.90 ± 14.63	120.12 ± 18.74	< 0.001

DBP (mHg)	81.20 ± 9.83	77.23 ± 7.93	0.005

HOMA-IR	10.42 ± 15.44	3.62 ± 2.89	< 0.001

FBG (mg/dl)	114.8 ± 27.9	100.4 ± 28.6	< 0.001

Total cholesterol (mg/dl)	195.76 ± 42.37	198.85 ± 40.18	n.s.

HDL-cholesterol (mg/dl)	46.76 ± 10.89	56.34 ± 12.29	< 0.001

LDL-cholesterol (mg/dl)	116.97 ± 43.39	119.41 ± 41.19	n.s.

Triglycerides (mg/dl)	172.5 ± 95.2	103.1 ± 47.6	< 0.001

AST (IU/l)	26.5 ± 15.4	18.9 ± 5.6	< 0.001

ALT (IU/l)	37.7 ± 24.6	20.4 ± 10	< 0.001

GGT (IU/l)	45.5 ± 71.2	20.3 ± 14.7	< 0.001

Alkaline phosphatase (IU/l)	73.3 ± 27.4	61.7 ± 20.9	0.02

25(OH) vitamin D (ng/ml)	14.8 ± 9.2	20.5 ± 9.7	< 0.001

FLI	71.66 ± 25.25	30.25 ± 28.93	< 0.001

MS^ (%/n)	73/118	23/23	< 0.001

T2D^ (%/n)	39/63	39/63	< 0.001

NAFLD degree	I = 43% II = 39.2% III = 17.8%		

Results are shown as mean ± SD. Student's T test. ^Chi-square test applied. HOMA-IR, FBG, triglycerides, AST, ALT, GGT and alkaline phosphatase were considered as log-values in the analysis.

### NAFLD and 25(OH) vitamin D

Patients with NAFLD had reduced serum 25(OH) vitamin D levels compared with subjects without NAFLD who had mean values just above the threshold of 20 ng/ml (50 nmol/l) for normal vitamin D status [28] (14.8 ± 9.2 vs 20.5 ± 9.7 ng/ml, p < 0.001, OR 0.95, IC 95% 0.92-0.98).

The association between NAFLD and low 25(OH) vitamin D levels was independent from age, sex, triglycerides, HDL and FBG in the multiple logistic regression analysis (p < 0.005) (Table 2) and is not affected by BMI in a multivariate logistic model comprising sex, age, 25(OH) vitamin D and BMI (25(OH) vitamin D: OR 0.93, C.I. 0.88-0.99, p = 0.02; BMI OR 4.4, C.I. 2.2-8.9, p < 0.001).

**Table 2:** Multiple logistic analysis.

Term	Odds Ratio	**95% C.I**.		Coefficient	**S. E**.	Z-Statistic	P-Value
Sex	1.28	0.54	3.02	0.25	0.44	0.56	0.57
Age	1.04	0.99	1.09	0.04	0.02	1.56	0.12
FBG	1.01	0.99	1.03	0.01	0.01	1.66	0.09
HDL	0.99	0.96	1.02	-0.01	0.02	-0.57	0.57
Triglycerides	1.01	1.00	1.02	0.01	0.003	2.75	0.0059
Vitamin D	0.93	0.88	0.98	-0.07	0.02	-2.80	0.0051
CONSTANT	*	*	*	-3.14	1.99	-1.58	0.11

NAFLD dependent variable.

FBG, fasting blood glucose; HDL, high density lipoprotein

P-values < 0.05 were considered significant. Likelihood Ratio < 0.001.

In our study population, we identified a subgroup of normal-weight subjects (n = 70, BMI< 25 kg/m^2^) in which the prevalence of NAFLD was 13.7%; also in this group of normal-weight individuals those with NAFLD had serum 25(OH) vitamin D levels significantly reduced compared with subjects without NAFLD (14.6 ± 9.7 vs 23.2 ± 8.9 ng/ml, p = 0.01). Multivariate regression analysis performed in this normal-weight population showed that the association between NAFLD and vitamin D was independent from sex, age, FBG, triglycerides and BMI (Table 3).

**Table 3:** Multiple logistic regression analysis in subject with BMI< 25 Kg/m^2^.

Term	Odds Ratio	**95% C.I**.		Coefficient	**S. E**.	Z-Statistic	P-Value
Sex	1.53	0.69	3.37	0.43	0.40	1.06	0.29
Age	0.97	0.84	1.12	-0.03	0.07	-0.42	0.68
FBG	1.00	0.95	1.06	0.01	0.02	0.19	0.84
HDL	0.88	0.12	6.14	-0.13	0.99	-0.13	0.89
Triglycerides	0.99	0.97	1.02	-0.01	0.01	-0.48	0.63
Vitamin D	0.86	0.76	0.98	-0.15	0.06	-2.33	0.019
CONSTANT	*	*	*	-5.90	9.14	-0.64	0.52

NAFLD dependent variable.

P-values < 0.05 were considered significant.

FBG, fasting blood glucose; HDL, high density lipoprotein

Moreover, we performed an ordinal regression analysis that showed a significant association between NAFLD scores, serum 25(OH) vitamin D levels, the components of MS and the insulin resistance degree, measured by means of HOMA-IR (Table 4).

**Table 4:** Ordinal regression analysis of factors associated with NAFLD scoring.

	SE	P-value
25(OH) vitamin D	0.01	0.001
Age	0.01	< 0.001
Sex	0.18	n.s.
BMI	0.03	< 0.001
Waist circumference	0.01	< 0.001
FBG	1.5	< 0.001
Total cholesterol	0.003	n.s.
HDL-cholesterol	0.01	< 0.001
LDL-cholesterol	0.003	n.s.
Triglycerides	0.7	< 0.001
HOMA-IR	0.8	< 0.001
Fasting insulin	0.6	< 0.001

SE, standard error of β. P-values < 0.05 were considered significant. HOMA-IR, FBG and triglycerides, were considered as log-values in the analysis.

FBG, fasting blood glucose; HDL, high density lipoprotein; LDL, low-density lipoprotein; HOMA-IR, homeostatis model assessment of insulin resistance.

After stratifying the study sample according to serum 25(OH) vitamin D quartiles, we observed an increased prevalence of NAFLD, MS and its components in the lowest 25(OH) vitamin D quartiles, whereas the prevalence of type 2 diabetes (T2D) was similar throughout the quartiles of vitamin D. Table 5 shows clinical and biochemical values of study population according with 25(OH) vitamin D quartiles and the results of inter-groups trend test.

**Table 5:** Clinical-biochemical characteristics of study population according to serum 25(OH) vitamin D quartiles.

	I Quartile (n = 51)	II Quartile (n = 66)	III Quartile (n = 62)	IV Quartile (n = 83)	P-value
Age (years)	55.93 ± 9.34	52.1 ± 10.7	52.2 ± 11.2	52.24 ± 9.45	n.s.
Sex° (M/F)	30/21	32/34	33/29	37/46	n.s.
BMI (kg/m^2^)	30.99 ± 6.96	29.5 ± 5.8	27.04 ± 4	26.25 ± 4.38	< 0.001
Waist circumference (cm)	105.27 ± 16.82	103.6 ± 14.8	95.01 ± 19.2	91.08 ± 16.72	0.001
FBG (mg/dl)	111.5 ± 26.1	106.3 ± 23.7	107.7 ± 30.2	103.2 ± 31.1	n.s.
Total cholesterol (mg/dl)	206.05 ± 49.46	201.3 ± 41.1	197.5 ± 35.8	192.07 ± 40.95	n.s.
LDL-cholesterol (mg/dl)	122.73 ± 44.62	126.8 ± 37.5	123.6 ± 33.2	109.15 ± 46.07	n.s.
HDL-cholesterol (mg/dl)	51.88 ± 16.36	52.5 ± 12.7	52.5 ± 12.2	52.71 ± 13.82	n.s.
Triglycerides (mg/dl)	178.8 ± 98.7	124.8 ± 84.7	124.6 ± 75	115.6 ± 60.8	0.001
HOMA-IR	10.01 ± 7.58	5.15 ± 4.3	4.3 ± 9.8	5.32 ± 7.86	0.03
AST (IU/l)	21.1 ± 7.4	22.1 ± 11.1	25.6 ± 10.7	21.8 ± 11.01	n.s.
ALT (IU/l)	25.5 ± 14	28.8 ± 16.6	28.6 ± 21.7	26.7 ± 19.2	n.s.
FLI	71.77 ± 26.83	51.9 ± 33.3	45.02 ± 35.9	32.04 ± 29.79	< 0.001
NAFLD ° (%)	68.3	56.1	50	37.3	0.01
MS ° (%)	72.5	43.9	35.5	33.3	< 0.001
T2D ° (%)	36.2	22.7	24.2	25.3	n.s.

Results are shown as mean ± SD. ANOVA test applied. Kruskal-Wallis test applied. HOMA-IR, FBG, triglycerides, AST and ALT were considered as log-values in the analysis. P-values < 0.05 were considered significant.

BMI, body mass index; FBG, fasting blood glucose; LDL, low-density lipoprotein; HDL, high density lipoprotein; HOMA-IR, homeostatis model assessment of insulin resistance; AST, aspartate aminotransferase; ALT, alanine aminotransferase; FLI, fatty liver index; NAFLD, non-alcoholic fatty liver disease; MS, metabolic syndrome; T2D, type 2 diabetes.

Interestingly, the lowest 25(OH) vitamin D quartile showed an OR = 4.71 (CI 2.15-10.3, p < 0.001) for NAFLD compared to the highest one.

### FLI, fatty liver and 25(OH) vitamin D

NAFLD patients had significantly higher FLI compared to non-NAFLD subjects (71.6 ± 25.2 vs 30.2 ± 28.9, p < 0.001) and the correlation between US detected NAFLD and FLI was extremely tight, both when considering fatty liver as dichotomous variable (r = 0.61, p < 0.001), and when considering NAFLD severity scale (r = 0.66, p < 0.001). FLI inversely correlated with 25(OH) vitamin D regardless sex, age and HOMA-IR (Unstandardized β coefficient: -1.6, standardized β coefficient: -0.4, p < 0.007).

### T2D and 25(OH) vitamin D

We also analyzed our study population according to the presence of T2D. Patients affected by T2D had serum 25(OH) vitamin D levels similar to non diabetic patients (17 ± 10.2 ng/ml vs 17.5 ± 8.8, p = n.s.); the logistic regression analysis performed in the whole population confirmed that vitamin D was not a determinant of diabetes (p = n.s.)

## Discussion

This study demonstrates that subjects affected by NAFLD have reduced serum 25(OH) vitamin D levels compared to age and sex matched individuals without NAFLD. This relationship is independent from the presence of T2D, MS and its individual components. Subjects belonging to the lowest vitamin D quartile display a significantly increased prevalence of NAFLD and MS, suggesting the presence of an overall increased cardiometabolic risk profile.

Previously, an association between low 25(OH) vitamin D levels and the histological severity of NASH was suggested by Targher et al. [29] in patients with chronically elevated liver enzymes and hepatic steatosis detected by US, who underwent liver biopsy for suspected steatohepatitis.

The present study was designed to investigate the relationship between fatty liver and hypovitaminosis D in a cohort of subjects with different degrees of insulin-resistance and no previously diagnosed liver disease, who were well characterized with respect to medical history, anthropometric measures and biochemical parameters.

NAFLD was assessed by liver US, which has been demonstrated to have a sensitivity of 83% and a specificity of 100% using histological criteria as gold standard [24]. US is suitable for routine evaluation of fatty liver in dysmetabolic patients, who, in most cases, do not get progressive liver disease and can be well managed without a need for liver biopsy, which cannot be performed at large in patients with no significant or trivial liver disease, mainly for ethical reasons. Yet, in our series, subjects had normal ALT and no clinical indication for histological confirmation of NAFLD.

We also calculated the Fatty Liver Index (FLI), a simple and accurate predictor of hepatic steatosis in the general population [25]. In our study cohort FLI tightly correlated with the presence/degree of NAFLD detected by US and the association between FLI and low vitamin D concentration was independent from sex, age and insulin resistance, quantified by means of HOMA-IR.

Vitamin D is known to be stored into the adipocytes and serum 25(OH) vitamin D levels could be significantly influenced by body composition. We did not make direct measurements of body fatness but we measured waist circumference and BMI which are proxies for adiposity, as largely demonstrated [30]. Because of the possible confounding role of adiposity in determining serum 25(OH) vitamin D levels among patients with BMI above normal limits [31], we performed a logistic multivariate analysis also adjusting for BMI demonstrating that the association between NAFLD and 25(OH) vitamin D persists after BMI adjustment. Furthermore, we performed a sub-analysis in normal-weight patients and controls and demonstrated that 25(OH) vitamin D was significantly reduced in NAFLD individuals compared to subjects without NAFLD, independently from fatness and other possible confounding factors.

The role of vitamin D in the pathogenesis of hepatic diseases is actually of great interest. In the liver, vitamin D acts as an "immune-modulator" suppressing fibroblast proliferation and collagen production [32,33].

Novel studies demonstrated that vitamin D deficiency was associated with low rate of sustained virological response (SVR) in patients affected by hepatitis C virus (HCV) under interferon-alfa therapy [34,35]. Furthermore, a recent intervention trial showed that vitamin D supplementation improves the probability of achieving a SVR following antiviral treatment in patients with recurrent hepatitis C [36].

Serum vitamin D inversely associated with the presence of dysmetabolic conditions in our study as well as in other published reports [5-7] and may play a role in both NAFLD and cirrhosis outcomes though its anti-inflammatory and insulin-sensitizing activities [37,38].

Moreover, vitamin D directly regulates the metabolism of FFAs by means of its action on peroxisome proliferator-activated receptor gamma (PPAR**-**γ) improving FFA-induced insulin resistance *in vitro*. Therefore, under condition of vitamin D deficiency, the increased FFAs flow in the bloodstream may promote fat storage into the liver and facilitate the development of NAFLD.

Our study has some limitations. First, the presence of less common causes of liver disease, such as autoimmune hepatitis, hemochromatosis, or Wilson's disease, cannot be ruled out in our patients. Second, although US is a practical approach commonly used to detect liver steatosis, it is not the gold standard technique for quantitative liver fat assessment. Another limitation of this study relates to its cross-sectional design, that does not allow to establish a causality nexus between low serum 25(OH) vitamin D levels and the presence of NAFLD.

## Conclusions

In conclusion, we demonstrate a strong independent association between low 25(OH) vitamin D levels and NAFLD in a population of adults without signs of severe liver damage even when the diagnosis of fatty liver is based on routine US examination; this association is independent from diabetes, lipid profile alterations and insulin resistance and is partially hidden by fatness in overweight subjects. Besides, an inverse correlation between serum 25(OH) vitamin D levels and the degree of NAFLD was observed, suggesting that vitamin D may exert a dose-dependent effect on fat accumulation into the hepatocytes.

Despite the cross-sectional design of this study, not allowing to establish a causal nexus, overall our data may suggest a significant contribution of hypovitaminosis D in the pathogenesis of liver steatosis. Intervention trials are warranted to evaluate whether vitamin D supplementation may be a means to prevent and/or treat NAFLD.

## Abbreviations

ALT: alanine aminotransferase; AST: aspartate aminotransferase; BMI: body mass index; FBG: fasting blood glucose; FFA: free fatty acids; FLI: fatty liver index; gamma-GT: gamma-glutamyl transpeptidase; HCV: hepatitis C virus; HDL: high density lipoprotein; HOMA-IR: homeostatis model assessment of insulin resistance; LDL: low-density lipoprotein; MS: metabolic syndrome; NAFLD: non-alcoholic fatty liver disease; NASH: non-alcoholic steatohepatitis; NCEP/ATPIII: Third Report of National Cholesterol Education Program/Adult Treatment Panel; PPAR**-**γ: peroxisome proliferator-activated receptor gamma; T2D: type 2 diabetes.

## Competing interests

The authors declare that they have no competing interests.

## Authors' contributions

All authors were involved in the conception and design of this study. IB, FA, MDB and MGC collected study population and performed clinical and laboratory evaluations. IB performed clinical and laboratory data collection. IB, FA, MGB and MGC wrote the manuscript, performed statistical analysis and organized data presentation. SM and PP provided intellectual contribution to the manuscript. MGC coordinated the study. All authors revised the article critically for important intellectual content and gave final approval of the version to be published.

## Pre-publication history

The pre-publication history for this paper can be accessed here:

http://www.biomedcentral.com/1741-7015/9/85/prepub
